# General Practitioners’ responses to global climate change - lessons from clinical experience and the clinical method

**DOI:** 10.1186/1447-056X-11-6

**Published:** 2012-08-08

**Authors:** Grant Blashki, Alan Abelsohn, Robert Woollard, Neil Arya, Margot W Parkes, Paul Kendal, Erica Bell, R Warren Bell

**Affiliations:** 1 Nossal Institute for Global Health & The Melbourne Sustainable Society Institute, The University of Melbourne, 161 Barry St, Carlton, Melbourne, 3010, Australia; 2 Department of Family and Community Medicine and Dalla Lana School of PublicHealth, University of Toronto, 1466 Bathurst Street, #205, Toronto, ON, M5R-3S3, Canada; 3 Department of Family Practice, University of British Columbia, 320-5950University Blvd, Vancouver, BC, V6T 1Z3, Canada; 4 Director Global Health Office Western University AssistantClinical Professor Family Medicine McMaster University and Adjunct Professor Environment and Resource Studies University of Waterloo, 200 University Avenue West, Waterloo, ON, N2L 3G1, Canada; 5 School of Health Sciences, University of Northern British Columbia, 3333 University Way, Prince George, V2N 4Z9, Canada; 6 Rural Coordination Centre of British Columbia, University of British Columbia, 300 - 5950 University Blvd, Vancouver, BC, V6T 1Z3, Canada; 7 University Department of Rural Health, University of Tasmania, Elizabeth St, Hobart, 7001, Australia; 8 Canadian Association of Physicians for the Environment Active Staff, Shuswap Lake General Hospital Salmon Arm, Salmon Arm, BC, V1E 4S2, Canada

## Abstract

**Background:**

Climate change is a global public health problem that will require complex thinking if meaningful and effective solutions are to be achieved. In this conceptual paper we argue that GPs have much to bring to the issue of climate change from their wide-ranging clinical experience and from the principles underpinning their clinical methods. This experience and thinking calls forth particular contributions GPs can and should make to debate and action.

**Discussion:**

We contend that the privileged experience and *GP way of thinking* can make valuable contributions when applied to climate change solutions. These include a lifetime of experience, reflection and epistemological application to ***first doing no harm***, ***managing uncertainty***, the ability to ***make necessary decisions while possessing incomplete information***, an appreciation of ***complex adaptive systems***, maintenance of ***homeostasis***, vigilance for ***unintended consequences***, and an appreciation of the importance of ***transdisciplinarity and interprofessionalism.***

**Summary:**

General practitioners have a long history of public health advocacy and in the case of climate change may bring a way of approaching complex human problems that could be applied to the dilemmas of climate change.

## Background

The history of humanity is a story of a species populating the world from its origins in Africa, and surviving climactic challenges through extraordinary ingenuity and tenacity. It’s clear that Homo Sapiens needed to adapt to major climatic changes over tens of thousands of years as they sought ways to guarantee their food supply, initially as nomads, and then later through cultivating the land. What is unique about this time in history is that we are witnessing human induced pressures on the Earth’s life support systems at a rate and scale that challenges the capacity of even robust ecosystems to adapt quickly enough.

The leading scientific academies around the world are in agreement that the average global temperature is rising, and that it is most likely mainly attributable to human activities, in particular the emission of greenhouse gases [1]. While some legitimate debate continues regarding the extent, rate of human contributions to warming, the most respected journals and scientific institutions are confident that climate change is indeed real and is a serious concern for future generations [2-5].

The impacts of climate change are already observable in a range of physical and biological systems [6]. The list includes; more frequent and severe heatwaves [7], changes in precipitation which results in increased flooding in some regions and drought in others [1], increases in the rate of sea level rise [1], an increase in the strength of storms and other extreme weather events [1], an overall pattern of glacial retreat [1], and alterations in the distribution of infectious diseases including water and vectorborne diseases [8]. A number of plant and animal species are exhibiting changes in their geographical distribution and the changes to the timing of their life cycles are consistent with a global warming signal [6].

All these changes can have significant impacts on public health [9]. Some of these effects are direct such as injuries from extreme weather events [8], or increased mortality and morbidity related to heatwaves [7]. There are other impacts that are less direct such as the effects of warming on food security [10], clean water [11,12], environmental refugees [13], and the mental [14] and economic [15] well-being of communities who are subject to climate change effects. Together, climate change and its effects are recognised as one of the great threats to global public health in the coming century [16].

## Discussion

### The role of General Practitioners

General practitioners are first and foremost concerned with the enormous task of providing competent compassionate clinical care of the patient who presents to them [17]. GP training and clinical experience tends to lead to a strong focus on individuals attending for consultations, usually ahead of the public health perspective [17]. It is also true to say that the traditional general practitioner is not usually a key decision maker when it comes to making societal policy decisions that will dramatically affect climate change, for example, the way in which we obtain our energy supply, our foreign trade policies, or even our societal patterns of consumption.

Yet there is much that general practitioners can offer. They have a special role as the generalists of the medical system who bring a broad systems perspective and an understanding of their local communities [18]. From a practical perspective there is much that general practitioners can do to help encourage mitigation of climate change [19] and also to assist communities in their adaptation efforts [20]. A clear example is the provision of health care in an environmentally responsible manner, for example, programs in Australia and New Zealand which assist practices to reduce energy, waste and water usage [19,21] General practitioners can also use their relationships with their patients to encourage lifestyles that are both good for the patient’s health and also good for the environment, for example encouraging active transport, and eating locally grown fresh food [22]. Additionally GPs can play a role in advocacy for strong mitigation targets within their countries, and this can be achieved individually or through joining a range of advocacy groups nationally, or internationally, such as the International Society of Doctors for the Environment [23,24], the International Association for Ecology & Health, the environmental working group of WONCA [25] , WHO [26] and other global medical organizations.

The regional nature of climate change, which is about interactions between place, community and individual health mean that general practitioners have a unique perspective about the health impacts of climate change in their local regions that national and international level data does not capture so well.

All these activities are worthwhile and important contributions that general practitioners can make. However the remainder of this paper is more conceptual, exploring how the perspective and culture of general practice, with the immense experience of literally hundreds of thousands of patient visits over a lifetime, might be applied to the *complex* problem climate change presents (see Table 1).

**Table 1:** Application of GP clinical principles to climate change dilemmas

**GP axiom**	**Clinical application**	**Climate change application**
*First do no harm*	Consider potential risks of all clinical management plans	Ensure responses and solutions to climate change don’t do harm
*Manage Uncertainty*	Utilise probabilistic decision making in relation to uncertain diagnosis or management	Undertake major societal decisions in context of scientific uncertainty about climate change predictions
*Appreciate Complex Adaptive Systems*	Understand the interconnectedness of human biological systems	Appreciate the interdependence of geological, atmospheric, water and food systems
*Maintain homeostasis*	Recognise the prominence of balance as a cornerstone of human wellbeing	Recognise the balance of earth systems as an indispensable requirement for supporting life
*Value generalism*	Apply a broad psychosocial approach to maximise patient wellbeing	Don’t lose sight of the “big picture” in regards to climate change and recognise the need for contributions from multiple disciplines

### First do no harm

The medical principle of *Primum non nocere* arose at a time when the understanding of anatomy and physiology was rudimentary and physicians were wisely called upon to take no action unless there was a reasonable prospect of improving a patient’s problem. Similar wisdom can be applied to action on climate change. The principle is self-explanatory but often forgotten in medicine where too often the cure is worse than the disease. GPs well know that whenever managing a patient we need to weigh up the risks and benefits of tests and treatments against the illness itself. Far from a call to therapeutic nihilism it is a call to humility in suggesting interventions, considering their ramifications as far as can be determined and taking no irreversible action without considering risks, benefits and ***who*** is assuming each. This is a profoundly complex ethical process that has analogies in society’s approach to complex challenges in the ecosystem.

As societies seek solutions to climate change there are many potentially harmful wrong turns that could be taken. Perhaps the greatest risk is that in addressing climate change, the vast inequities in the world (with their attendant impact on everyone’s health) are further exacerbated [27]. Great care is needed so as not to further economically burden the poorest and most vulnerable people in the world. Choices about new forms of energy are also fraught with technical pitfalls. Nuclear energy may arguably well be less carbon polluting to the atmosphere (at least excluding uranium mining and the uncertain costs of disposal of waste) but the risks of misuse of nuclear materials is perhaps the only global threat which is on par with climate change itself. How nations choose to adapt to some inevitable impacts of climate change can also potentially exacerbate the problem–for example, mass air conditioning powered by carbon polluting energy sources to deal with heatwaves and warmer temperatures may provide some short-term relief but clearly is not in our long-term interests. The principle of ***Primum non nocere*** is a valuable guide to wisdom and safety in this area.

### Managing uncertainty

General practitioners are no strangers to uncertainty–undifferentiated illness, early presentations and diagnostic ambiguity are our stock in trade. So for example, a patient presenting with back pain could mean a wide range of possibilities including a minor strain, a metastasis of a cancer, or even psychological distress. Though it is true that managing uncertainty in healthcare is not the same as uncertainty in climate science, the basic skills are transferable. Interpreting small changes in the average global temperature of the planet and the follow-on effects in physical and biological systems requires the same probabilistic decision-making that GPs are so accustomed to in interpreting early signs of disease in their patients. Take for example hypertension, a sign invisible to the patient but detectable by the clinician and one that heralds potentially catastrophic ramifications in the future such as a stroke or blindness. Even a raised temperature, and its significance for the human body, is metaphorically and literally analogous to the rising temperature that climate scientists now detect in our atmosphere.

To make decisions in the context of uncertainty and to properly assess risks and benefits of courses of action (or inaction) is a great strength that the discipline of general practice can bring to issue of climate change. In their communities general practitioners are ideally placed to offer nuanced approaches to uncertainty and risk management in a context where even scenario modelling is likely to have major limitations. For example, consider the management of malaria, which has proved to be a major challenge for regional forecasters, precisely because of the way that local contexts (e.g. the presence of preventative activities and services) can mediate health effects [28-30].

### Appreciation of complex adaptive systems

The importance of understanding *complex adaptive systems* resides in recognising their intrinsic unpredictability, a property appreciated by GPs, and one that is crucial to appreciating our climate system. A complex adaptive system is a dynamic network of interconnected agents that impact on each other, and which results in *emergent properties* of the whole system. Emergent properties can best be understood as that point when a system, such as a sports team, becomes *more* than the sum of its parts. Such systems, which include most living things, are open and non-linear which means small effects can have great ramifications for the whole system. This study of complexity has been used to understand diverse systems such as the workings of the brain, the behaviour of insects and even the machinations of the economy.

It can be helpful to differentiate between simple, complicated and complex approaches to problem solving [31]. Simple problems can be dealt with by a recipe properly applied. However the differentiation between *complicated* and *complex* problems is a more nuanced yet fundamental difference to understand when considering global warming and the specific skills the GP can bring to the issue. Compare for example a *complicated* problem, (lets say, sending a rocket to the moon), with a *complex* problem, (for instance raising a child). In the former example, current efforts inform future attempts, formulae are essential, high levels of expertise in multiple fields are necessary and rockets are similar in critical ways. However, in the case of raising a child, formulae have a limited application, raising one child provides experience but not assurance with the next, expertise is necessary but not sufficient for success and every child is unique and must be understood as an individual.

As general practitioners we have a built-in sense of this type of complex thinking, and we see it in action on a daily basis in clinical situations- from the unexpected side-effects and interactions of medications through to the psychological reverberations of trauma through families and communities. Every day we see medical conditions play out in unpredictable and often unexpected ways as patients, their families and communities adapt (or don’t) to challenges to their health. An apparently small dysfunction can spiral a patient into high levels of distress and disability. And seemingly catastrophic illness can unexpectedly resolve through healing mechanisms not always apparent to us. It is a rare day in practice when we are not confronted with both the fragility and the resilience of human flesh and the human experience.

So too the emergent properties of the climate are driven by processes, which characterise complex adaptive systems. For example, a slightly warmer atmosphere can drastically increase its capacity to carry moisture, resulting in shifts in the patterns of precipitation–sometimes causing floods, sometimes causing droughts. And, as with human physiology, there are tipping points where, the capacity of the system to adapt is overwhelmed. The great risk of climate change is that it may tip the climate system, like a patient going into renal failure, into an irreversible new state. For instance, climate change could trigger a positive feedback loop where melting permafrost releases long trapped methane gases that in turn further warm the planet.

In the same spirit of exploration with which the generalist physician approaches complex disease, they can set aside the debilitating hopelessness often attending complex and large scale change, and thoughtfully seek to build interventions and feed-back loops that are characteristic of solutions to complex problems. Adaptive expertise is the hallmark of general practice and it is also a key area where climate change and health research and medical education, training and practice have much to learn from each other [32]. The adaptive expertise of general practitioners stands in contrast to the approach to complicated problems that demands mechanistic interventions and predictable, reproducible impacts from experimental interventions.

### Maintenance of homeostasis

Life is a balancing act, from human physiology through to the Earth’s physical systems that allow life to flourish. It is clear that the earth, like the human body, has numerous regulatory systems, and negative feedback loops which like the porridge in the Goldilocks and the Three Bear’s fairytale is “just right” for life [33]. Our bodies have evolved complex hormonal and chemical thermostats to make sure our temperature, our salts, and our fluids all stay within a narrow range necessary for life. Every patient encounter and proposed therapy must take this *homeostasis* into account. It is the basis for the fragility and resilience noted above.

Similarly, the Earth’s physical systems maintain the concentrations of key elements in our oceans and atmosphere and the lands that allow life to exist and any approach to influencing them must humbly take this into account. For example, concentrations of carbon dioxide in the atmosphere (the natural greenhouse) are crucial to keeping the Earth within a temperature range in which human beings can live and even thrive. However, all homoeostatic systems have their limits and we can see the impacts of excessive carbon dioxide beyond that which the oceans can absorb, resulting in ocean acidification with catastrophic implications for shell formation and the oceans’ food chain [34]. It is not simply that general practitioners understand homeostasis: through their special relationship with their local communities, they have intimate knowledge of the factors that shape fragility and resilience in those communities.

### Generalism

General practitioners are generalists–they are not constrained by the demographics of their patients, nor the nature of their illnesses [35]. As non specialists GP's play a key role in integrating health care and pulling together the skills of their specialist colleagues, as well as other types of knowledge, to improve service for patients and foster healthier communities [35].

The approach to the problem of climate change requires a similar balance of specialisation and generalism. What is required is problem solving at multiple levels, paralleling the role of the GP, to tie together the strands of knowledge from experts from different fields –from climate scientists to economists to psychologists just to name a few professions; and from the values and aspirations of people and communities themselves.

### Summary

It is clear from the failures of the world to act on climate change that scientific knowledge alone is necessary but not sufficient to create political climate change solutions. Lessons from general practice, which is often the common sense of the health system, could be usefully applied to bringing together society wide transformations that effectively address climate change.

## Competing interests

The Author declare that they have no competing interest.

## Author contributions

GB conceptualised the initial manuscript. All authors have contributed to the conceptual development and commented on successive drafts of the manuscript.
